# COVID-19 as cause of death: a bridge coding study

**DOI:** 10.3205/id000100

**Published:** 2025-12-10

**Authors:** Peter Harteloh

**Affiliations:** 1 The Hague, The Netherlands

**Keywords:** COVID-19, cause of death, cause-of-death statistics, death certificate, bridge coding study, ICD-10

## Abstract

**Background:**

The number of COVID-19 deaths is an important measure for the impact of the pandemic. However, estimates differ and fuel the debate on COVID-19 as legitimate cause of death.

**Objective:**

To study the role of COVID-19 as cause of death.

**Methods:**

Double (bridge) coding of all death certificates mentioning COVID-19 in the Dutch cause-of-death registry during the pandemic 2020–2022 (n=51,288). The coding of records by the WHO special instruction for COVID-19 as issued in April 2020 was compared with the coding of the same set of records by the prevailing rules of the ICD-10 and the effect on cause-of-death statistics was studied.

**Results:**

When mentioned on a death certificate, COVID-19 was selected as underlying cause of death in 94% of the cases by the WHO special instruction. According to the prevailing ICD-10 coding rules, COVID-19 was the beginning of a causal sequence leading to death in 76% of the cases (General Principle) and when the role of contributing co-morbidity was taken in to account too (Direct Sequel), COVID-19 was the underlying cause of death in 49% of the cases. These different estimates can be explained by a difference in perspective. The WHO special instruction identifies cases from an epidemiological point of view (surveillance), while the prevailing ICD-10 rules identify cases with COVID-19 as a necessary *and* sufficient cause of death from a medical (pathophysiological) point of view.

**Conclusion:**

Different estimates of COVID-19 deaths represent different views on the role of COVID-19 as cause of death, which should be taken in to account when interpreting cause-of-death statistics.

## Introduction

The number of deceased attributed to COVID-19 reflects the impact of the pandemic on global health [1]. However, estimates differ considerably and require an explanation [2]. The attribution of a death to COVID-19 is difficult and disputed [3], [4], [5], [6]. Nowadays, a death certificate reports on average three causes of death [7]. Selection is inevitable for assigning the death of a person to a (one) cause for statistics. The instruction manual of the ICD-10 provides rules for such a selection in order to obtain international comparable data for health policy purpose [8]. According to these instructions, cause-of-death statistics are a tabulation of *underlying causes of death*, one per deceased, defined as the starting point of the causal sequence leading directly to death ([8], p. 23). This is called the General Principle (GP) of cause-of-death statistics as the selected underlying cause of death should explain all the other causes reported on a death certificate. The GP is not only part of the ICD-10, but has a long tradition covering different previous versions of the International Classification of Diseases (ICD) as well [9]. In April 2020, WHO issued a special instruction prescribing to attribute death to COVID-19 in every deceased with a natural cause of death and COVID-19 being mentioned on part 1 of a death certificate [10]. This is a deviation from the GP as a causal sequence containing COVID-19, but not starting with COVID-19 is neglected. This study investigates the impact of this WHO special instruction on cause-of-death statistics by comparing its application with the prevailing ICD-10 rules on the same data set (bridge coding study) in order to better explain different estimates of COVID-19 deaths. 

## Method and material

### Method

A bridge coding study was performed, defined as processing the same set of death certificates by two different methods. Bridge coding studies are used for studying changes of classifications (e.g. ICD-9 versus ICD-10), the introduction of new coding methods (e.g. manual versus automated coding), or the introduction of new ICD-10 codes [11], [12], [13], [14]. At its outbreak in 2019, COVID-19 was a new disease. It brought new ICD-10 codes and principles for selecting COVID-19 as underlying cause of death. The WHO special instruction for selecting COVID-19 death states: “The primary goal is to identify all deaths due to COVID-19. A death due to COVID-19 is defined for surveillance purposes as a death resulting from a clinically compatible illness, in a probable or confirmed COVID-19 case, unless there is a clear alternative cause of death that cannot be related to COVID disease (e.g. trauma)” ([10], p. 3). And: “A death due to COVID-19 may not be attributed to another disease (e.g. cancer) and should be counted independently of preexisting conditions that are suspected of triggering a severe course of COVID-19” ([10], p. 3). This principle was incorporated in the Iris software for automated coding of death certificates by an update of September 2020, so that the WHO special instruction became part of the regular production of cause-of-death statistics in the Netherlands. 

For this (bridge coding) study, all death certificates mentioning COVID-19 in 2020–2022 were re-coded by the prevailing ICD-10 coding principles. The General Principle (GP) was applied when COVID-19 was reported on the lowest used line in part 1 of the death certificate, regardless any other causes mentioned on part 2 of the death certificate. When other contributory causes of death were mentioned in part 2 of the death certificate the so-called Direct Sequel (DS) was applied. Disorders with a possible causal connection – dementia, diabetes mellitus, COPD, stroke or malignancies – were preferred as underlying cause of death despite their position on part 2 of the death certificate in the same way the ICD-10 instruction manual prescribed the DS for a pneumonia being a ”clinical compatible illness” of COVID-19 ([8], p. 29). The reason for applying the DS is that although from a medical point of view the (only) cause of pneumonia is a micro-organism, a pneumonia becomes a cause* of death* by another disease without which the person would not have died from the pneumonia (counterfactual). The DS is part of the regular ICD-10 coding principles, but was disregarded in case of COVID-19. 

Death certificates with COVID-19 on the lowest used line and no other causes mentioned require no selection. The outcome of the recoding cannot differ from that of the routine coding process for this kind of death certificates. From a causal point of view, these records represent deaths in which COVID-19 was considered the one and only cause. Such cases represent COVID-19 as a necessary (the patient would not have died without COVID-19) *and* a sufficient (no other conditions required for dying) cause of death.

For comparing the outcome of the recoding with the outcome of the routine coding process, two measures were used: the perfect compatibility percentage (PCP), i.e. the number of records that would have been coded in exactly the same way by the different coding principles, and the comparability ratio (CR), i.e. the ratio of the different outcomes of the different coding principles for estimates in statistics [11], [12], [13], [14].

In formula: 

PCP=((number of cases with COVID-19 as underlying cause of death both according to WHO special instruction *and* according to ICD-10 vol. 2 principles (yes/yes) + number of cases without COVID-19 as underlying cause of death both according to WHO special instruction *and* according to ICD-10 vol. 2 principles (no/no)) divided by the total number of cases mentioning COVID-19 on a death certificate) x 100.

CR=number of cases with COVID-19 as underlying cause of death according to WHO special instruction divided by the number of cases with COVID-19 as underlying cause of death according to ICD-10 vol. 2 principles.

### Study material

The material of this study comprised all death certificates mentioning COVID-19 during the pandemic (2020–2022) in the Netherlands (n=51,288). In the Netherlands, for every deceased a death certificates was issued by the attending physician reporting a causal chain of morbid events leading to death (part 1 of the death certificate) or (if applicable) reporting diseases contributing to death while not being part of the causal chain (part 2 of the death certificate) [8]. For readers not so familiar with death certificates Table 1 (Tab. 1) shows the ICD-10 prescribed format of a death certificate with examples of the different ways COVID-19 was reported in combination with other causes of death. Applying different selection principles lead to different numbers of COVID-19 being designated as underlying cause of death for statistics (Table 1 (Tab. 1)).

Death certificates were processed by Statistics Netherlands [15], [16]. All causes of death mentioned on a death certificate were coded and the underlying cause of death – the starting point of the causal sequence of morbid events that led to death – was selected. During the years 2020–2022, death certificates were automatically coded by Iris – software for coding causes of death and selection of an underlying cause of death – version 5.6 [17]. All death certificates mentioning COVID-19 were reviewed manually by medical coders before being processed by Iris in order to ensure COVID-19 was coded according to the WHO special instruction of April 2020. All other death certificates were coded by the rules and guidelines of the instruction manual (Volume 2) of the ICD-10 as incorporated in the software of Iris.

### Sample

The sample comprised of 45.9% women and 54.1% men. The mean age of death was 83.8 years for women and 80.3 years for men. Of the deceased with COVID-19, 61% died in a nursing home, 30% in a hospital and 9% at home. Of all COVID-19 diagnoses reported, 91.6% were clinical or laboratory confirmed (ICD-10: U07.1) and 8.4% suspected (ICD-10: U07.2). Deceased with an external cause of death (suicide, accident, violence) were excluded from the sample as the WHO special instruction and the prevailing ICD-10 rules did not differ for assigning the underlying cause of death to this kind of records [10].

## Results

**Figure 1:**
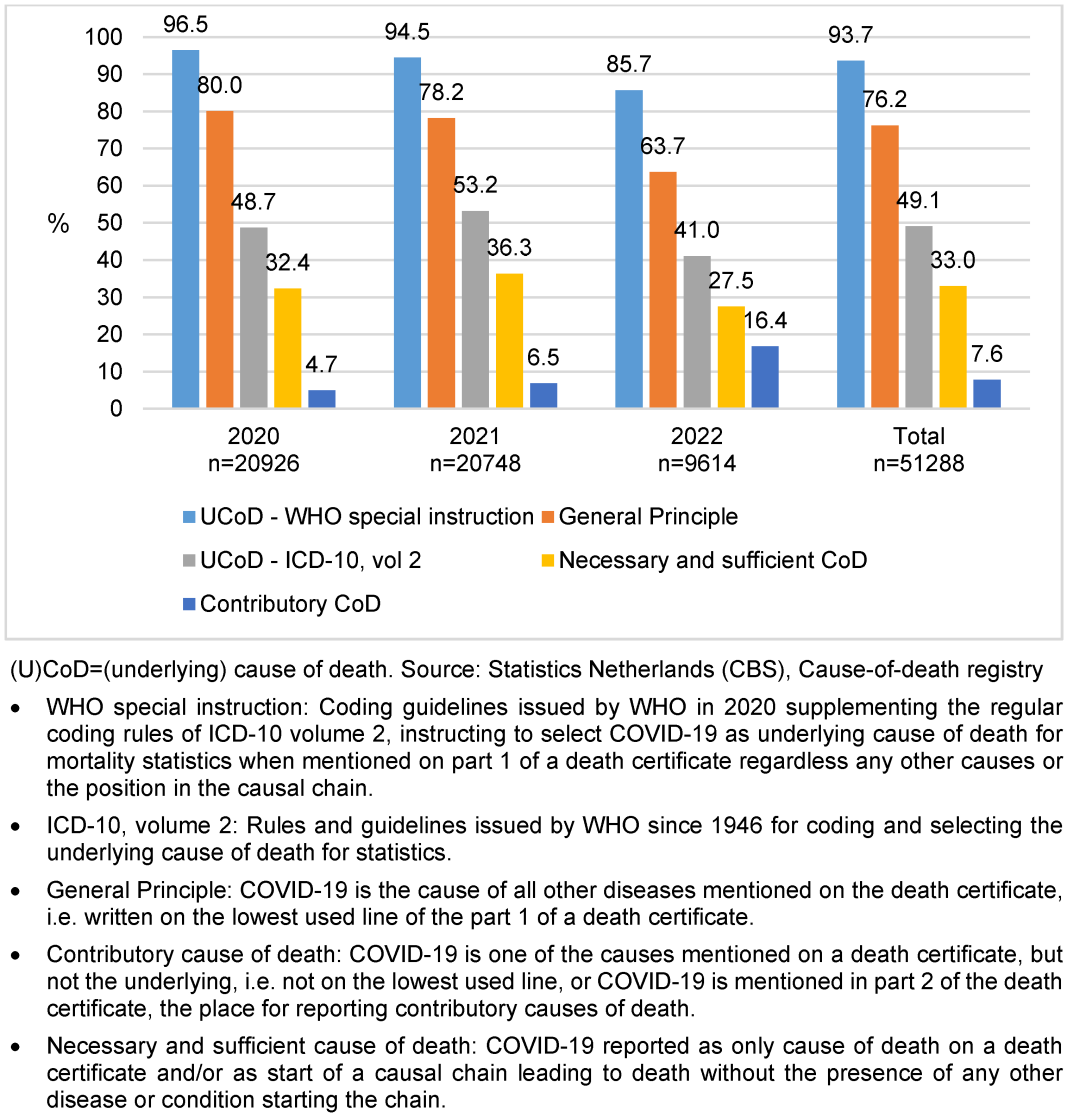
COVID-19 (confirmed and suspected) as cause of death by different coding principles, percentage of records mentioning COVID-19 above columns

Figure 1 (Fig. 1) shows the outcome of different coding principles applied to the same data set for each year of the pandemic. Of all death certificates mentioning COVID-19, the disease was designated as underlying cause of death for statistics by the WHO special instruction in 93.7% of the cases. In 76.2% of the cases the General Principle applied (COVID-19 start of causal sequence) and in 49.1% of the cases COVID-19 remained underlying cause of death when reported co-morbidity was taken in to account by applying the DS as instructed in volume 2 of the ICD-10. COVID-19 was reported on the lowest used line of a death certificate without any other causes, i.e. as necessary and sufficient cause of death, in 33.0% of all cases mentioning the disease. The percentage of cases designated to COVID-19 as underlying cause of death decreased in the course of the pandemic due to an increase of COVID-19 reported as contributory cause on part 2 of a death certificate: from 4.7% in 2020 to 16.4% in 2022 (Figure 1 (Fig. 1)). 

Comparability ratios (CR) follow from Figure 1 (Fig. 1). For the whole sample, the CR was 1.23 (93.7/76.2) with regard to the position of COVID-19 on a death certificate (General Principle) and 1.91 (93.7/49.1) when the direct sequel was applied. 

The perfect compatibility percentage (PCP) follows from Table 2 (Tab. 2), showing a crosstabulation of the number of cases with COVID-19 as underlying cause of death according to two different selection principles: the WHO special instruction for COVID-19 and the prevailing rules of the ICD-10 (volume 2) instruction manual. The PCP ((25,062+3,135)/51,288) was 55.0%. Almost all cases considered underlying cause of death from a medical point of view as represented by the ICD-10 instruction manual (25,062/25,173) were captured by the WHO special instruction too (sensitivity high). However, too many cases were designated as underlying cause of death by the WHO special instruction (48,042/25,173) (specificity low). 

## Discussion

This is a bridge coding study of death certificates mentioning COVID-19. The coding of death certificates by the WHO special instruction for COVID-19 was compared with a coding of the same set of death certificates by the prevailing rules of the ICD-10 (Volume 2) instruction manual. In about 94% of the cases mentioning COVID-19, the disease was selected as underlying cause of death for statistics by the WHO special instruction. In 76% of the cases mentioning COVID-19, it was reported as the beginning of the causal chain leading to death (General Principle of the ICD-10 instruction manual applied). With the occurrence of co-morbidity taken in to account (Direct Sequel rule of the ICD-10 instruction manual applied), COVID-19 remained underlying cause of death in 49% of the deceased cases. COVID-19 was reported as necessary and sufficient cause of death in 33% of the deceased cases, implying comorbid conditions being reported in 67% of the cases. The perfect compatibility between the WHO special instruction and the prevailing ICD-10 rules for selecting COVID-19 as underlying cause of death was 55%. 

Up to date, no other (published) bridge coding studies on COVID-19 are encountered in medical literature. Grippo et al. studied death certificates mentioning COVID-19 in 2020 and found it to be the underlying cause of death in 88% of cases [18]. This finding is in line with the finding of this study for the year 2020 (80%). Grippo et al. reported comorbidities in 72% of the death certificates, with little variation by age and gender [18]. A finding also in line with the outcome of this study (67%). The Italian study was conducted at the beginning of the pandemic (2020). This study covers the course of the pandemic showing a decrease of COVID-19 as underling cause of death and an increase of COVID-19 as contributing cause of death.

This bridge coding study also shows that in 49% of the cases COVID-19 remained the underlying cause of death when the role of contributory causes reported in part 2 of the death certificate was taken in to account. As there are no other bridge coding studies available yet, this estimate cannot be compared with other research. 

The different estimates of COVID-19 deaths by these different coding principles can be explained by a different purpose of classification on the one hand and by a different idea of causality on the other hand. The WHO special instruction is motivated by surveillance ([10], p. 3). Deceased dying *with* COVID-19 are monitored to inform about the course of the pandemic. The regular ICD-10 rules for assigning an underlying cause of death are bases on medical causal considerations, i.e. the observation by a physician of a chain of events in time being connected by a pathophysiological mechanism. The start of such a causal chain leading to death can be the object of an intervention (therapy or prevention) preventing death ([8], p. 23). Deceased dying *from* COVID-19 are identified. 

The WHO special instruction of April 2020 serving surveillance is an exception to the ICD coding rules captured by a few pages as part of the ICD-6 to a complete volume of rules in the ICD-10 serving medical causality [8], [9]. Such an exception is not new. It is also used for selecting influenza as cause of death ([8], p. 50), ([10], p. 8). However, the number of cases dying with influenza is even during an epidemic outbreak (about 0.3% of the deceased) much less than the number of COVID-19 deaths (11% of the deceased). So, the impact of the exception during the COVID-19 pandemic was large and the special WHO instruction led to an ambiguous interpretation of cause-of-death statistics. In the years 2020–2022, the majority of other underlying causes (89%) represented the start of a causal chain leading to death, while the most frequently occurring cause of death (11%), COVID-19, was selected when being mentioned on a death certificate regardless its role as cause of death. The estimate represents cases dying *with* and cases dying *from* COVID-19. 

Such a deviation from the prevailing ICD-10 rules hinders cause-of-death statistics. It causes shifts in statistics as other diseases reported on a death certificate with COVID-19 (mainly dementia and COPD) are not selected as underlying cause of death for statistics [2]. Time trends have to be repaired [11]. Also, the purpose of surveillance might be questioned as there are other registrations serving this purpose well, for instance the mandatory registration of infectious diseases showing the course of the pandemic or the registration of positive screening tests. 

As the name implies, cause-of-death statistics incorporate causal theory and classify the role of a disease in dying [2]. This qualification requires a medical point of view as pathophysiological mechanisms are the causal connecting elements between diseases or disorders being reported on a death certificate [19]. First of all, COVID-19 can be considered a necessary *and* sufficient cause of death when reported as the only cause on a death certificate or as start of the causal chain without additional co-morbidity. This appeared to be the case in 33% of the deceased cases. In 67% of the deceased cases there was a cooperation of COVID-19 with other diseases causing death. According to causal theory, COVID-19 is an insufficient, but necessary part of an unnecessary but sufficient (INUS) condition in these deceased cases [18]. This means the patient would not have died at that particular moment in time without attracting the virus [20]. However, comorbidity is required for dying. COVID-19 is a necessary part of a combination of diseases sufficient for causing death. As there is not a fixed combination of causes, each combination as such is a sufficient, but not a necessary cause of death. With regard to infectious diseases such as COVID-19, current cause-of-death statistics identify so-called INUS conditions underlying death, but do not provide a full description of such a condition. For this death certificates reporting other causes beside COVID-19 have to be studied [21].

In studying COVID-19 as part of an INUS condition, there appeared to be a statistically significant association between COVID-19 and dementia, Parkinson’s disease, COPD, pneumonia and diabetes mellitus [2]. Other studies confirm this role of co-morbidity in death [22], [23]. It is the immuno-senescence in case of neurodegenerative diseases and the change of ACE2 receptors in case of COPD or diabetes that causally connects them with COVID-19 [24], [25], [26]. It makes COVID-19 a part, not the start of a causal chain. It justifies the application of the DS. With these pathophysiological mechanisms, i.e. the GP and DS, taken in to account, the estimate of COVID-19 deaths would have been about half than currently reported by cause-of-death statistics. 

Because of the shifts of underlying causes of death by the appearance of a new disease like COVID-19, the overall mortality is (rightly) considered a good indicator for the impact of the pandemic at population level [27]. However, for the individual the question of the actual cause of his or her death remains. Therefore, causal theory cannot be avoided, should be applied carefully and be consistent over time in order to monitor trends and epidemics. For this, a deviation from the regular ICD-10 coding principles can better be avoided as the current study shows. Other sources are available and each source provides an answer to a different question. The overall mortality statistics inform about the impact of the epidemic at population level [27]. The mandatory registrations of infectious diseases inform about the spread and impact of validated COVID-19 diagnoses (infection (clinical/subclinical) → disease → recovery, disability or death) in populations [28]. The cause-of-death registration (should) inform about the actual role of COVID-19 in dying.

### Strengths and limitations

The strong point of this study is its material. The role of COVID-19 as cause of death is studied by death certificates. Different perspectives were identified and their impact on cause-of-death statistics explained. This provides new information for the often heated debate on the number of COVID-19 deaths. Different estimates represent different views on COVID-19 as cause of death [2]. With regard to age and sex, the sample of death certificates studied can be considered representative for COVID-19 deaths in high income countries. As there are no other bridge coding studies identified in literature yet, the findings in this study require replication to validate its outcome. 

The strength of this study is also its weakness. The notation of COVID-19 on a death certificate provides no information about the clinical severity of the disease. A positive test result or a fully developed clinical syndrome are not distinguished, and in accordance with the WHO special instruction both are coded (ICD-10: U07.1) and selected as underlying cause of death in the same way [10]. However, from the fact that 91% of the deaths involved in this study are nursing home residents or hospitalized patients, interpreting the notation of COVID-19 on a death certificate as a developed clinical syndrome is reasonable, but this remains to be confirmed by other studies (compare death certificates with clinical records). 

Also, a limitation of this study is the proper completion of a death certificate. The certifier will have to determine whether a patient *would* have died anyway regardless COVID-19 (counterfactual) and if so do not report COVID-19 as cause of death [29]. This is more difficult for a new disease like COVID-19 than for diseases with a known pathophysiological mechanism. Thus, underreporting of causes has to be considered. 

Over reporting has to be considered as well. Some death certificates report a positive COVID-19 test result. Sometimes this adds information to a reported pneumonia, but what is the meaning in case of a non-COVID-19 related disease? Also the ambiguous notation of “COVID-19 positive” was encountered. This could indicate a clinical developed disease or “just” a positive test result. As there was no check of death certificates by questioning the certifier due to the high work pressure during the pandemic, the coding of death certificates might be biased in an unknown number of cases by reporting a positive COVID-19 test result. 

Therefore, linking the cause-of-death registry to other registries is recommended. By linking the cause-of-death registry with clinical registrations, insight can be obtained in the validity of the diagnosis, the severity of a disease and the co-morbidity present at the end of life, so that the role of a disease as cause of death can be further investigated [30].

## Conclusions

Different estimates of COVID-19 deaths represent different views on the role of COVID-19 as cause of death. According to the WHO special instruction, COVID-19 is underlying cause of death in 94% of the deceased cases. Applying the prevailing ICD-10 rules, COVID-19 is assigned the role of underlying cause of death in 76% of the deceased cases with regard to its position on the lowest used line of a death certificate and in 49% of the deceased cases if the reported co-morbidity is taken in to account by the direct sequel rule of the ICD-10. The different estimates can be explained by causal theory. The WHO special instruction identifies cases dying *from* or *with* COVID-19 from an epidemiological point of view (surveillance), while the prevailing ICD-10 rules identify cases dying *from* COVID-19 from a medical (pathophysiological) point of view. These different perspectives should be taken in to account when interpreting the occurrence of COVID-19 in cause-of-death statistics. 

## Notes

### Acknowledgement

The author likes to thank an anonymous reviewer for critical reading and suggestions for improvement of the manuscript. No AI was used in producing the data or the manuscript. 

### Ethics

There were no living persons involved in this study. According to Dutch Civil Law (Article 7: 458) no ethical approval is required for a secondary analysis on non-identifiable data of deceased persons. 

### Short author biography

Peter Harteloh worked as medical epidemiologist on cause of death statistics for Statistics Netherlands, Section Health and Care, for 17 years (2007–2024). He is retired now.

### Author’s ORCID

Peter Harteloh: 0000-0001-6271-3813

### Disclaimer

The opinions expressed in this article are of the author and do not necessarily represent the views of Statistics Netherlands.

### Competing interests

The author declares that he has no competing interests.

## Figures and Tables

**Table 1:**
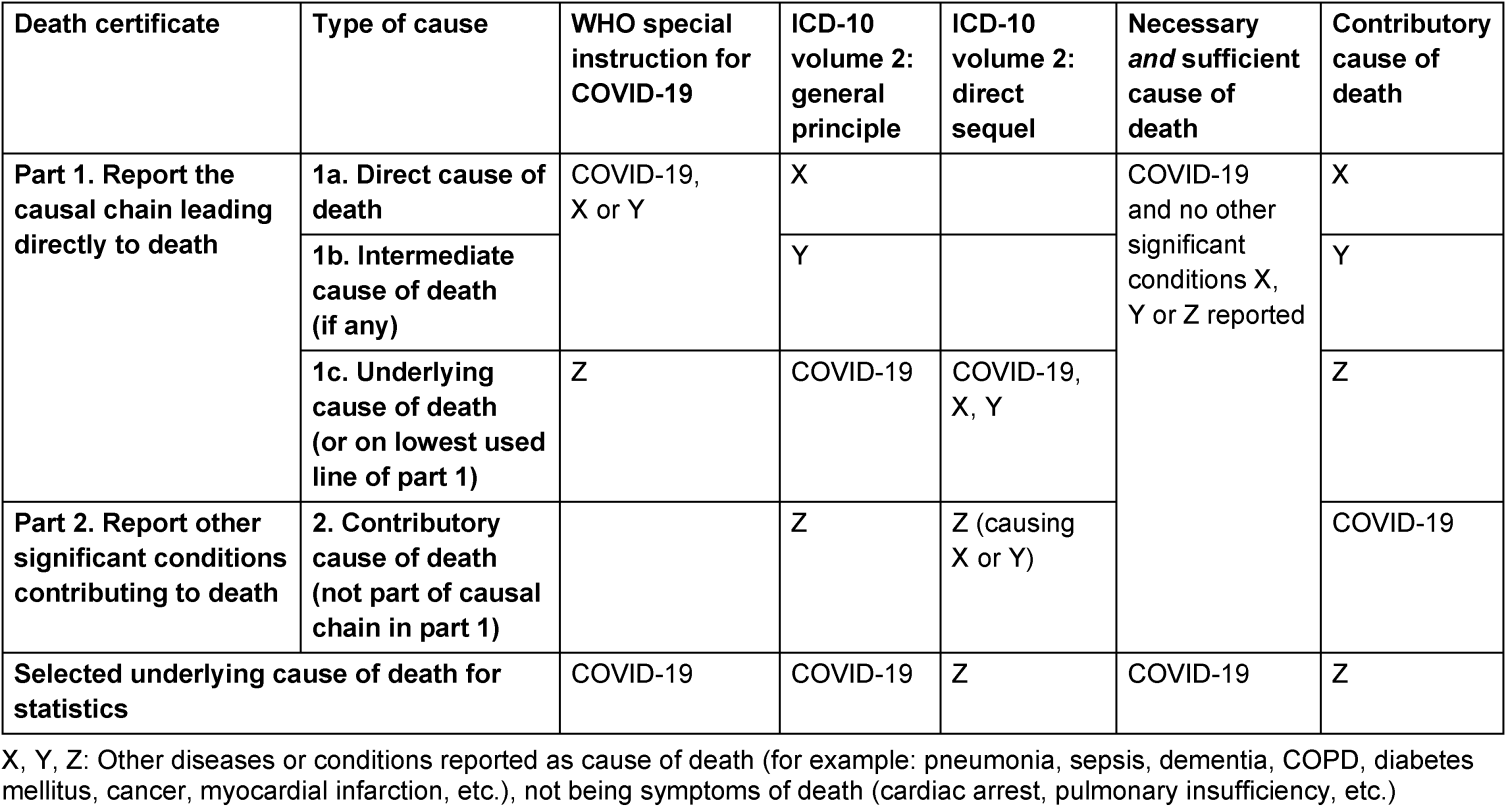
Reporting COVID-19 on death certificates

**Table 2:**
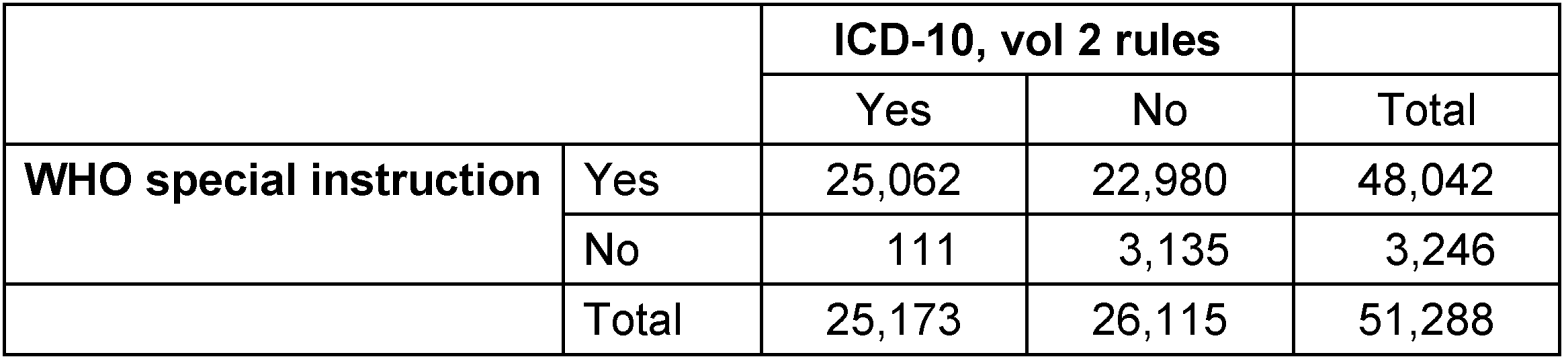
COVID-19 as underlying cause of death (2020–2022)
